# High gain, wide-angle QCTO-enabled modified Luneburg lens antenna with broadband anti-reflective layer

**DOI:** 10.1038/s41598-020-69631-6

**Published:** 2020-07-28

**Authors:** Soumitra Biswas, Mark Mirotznik

**Affiliations:** 1 Electrical and Computer Engineering Department, University of Delaware, Newark, DE 19716 USA; 2 Advanced Antenna Technology Group, Envistacom, Peachtree Corners, GA 30092 USA

**Keywords:** Engineering, Electrical and electronic engineering

## Abstract

The gradient-index (GRIN) Luneburg lens antenna offers significant benefits, e.g. high aperture efficiency, low-power, minimal cost, wide beam scanning angle and broad bandwidth, over phased array antennas and reflector antennas. However, the spherical shape of the Luneburg lens geometry complicates the integration of standard planar feed sources and poses significant implementation challenge. To eliminate the feed mismatch problem, the quasi-conformal transformation optics (QCTO) method can be adopted to modify the lens’ spherical feed surface into a planar one. However, Luneburg lenses designed with QCTO method are limited to poor performance due to the presence of the reflections and beam broadening arising from the quasi-conformal mapping. In this paper, we present a new method of implementing QCTO-enabled modified Luneburg lens antenna by designing a broadband anti-reflective layer along with the modified lens’s planar excitation surface. The proposed anti-reflector layer is inherently broadband in nature, has a continuously tapered inhomogeneous dielectric permittivity profile along its thickness, and ensures broadband impedance matching. To show the new QCTO modified Luneburg lens antenna, an example lens antenna was designed at Ka-band (26–40 GHz) and fabricated using fused deposition modeling (FDM) based additive manufacturing technique. Electromagnetic performance of the lens antenna was experimentally demonstrated.

## Introduction

Modern wireless communication systems are increasingly focusing on high gain, agile, wide-angle, multiband and multibeam beamscanning antenna elements for applications in radar, electronic warfare, wireless and satellite communication systems^1–4^. Conventionally, these properties have been exclusively achieved by using either electronically steerable phased array antenna technology or mechanically steerable reflector antenna systems. Electronically steered phased array antenna system is very sophisticated and agile,however, this technology requires all the antenna elements to be active simultaneously and utilizes phase shifters at every antenna element for electronic beam steering. These antenna elements and phase shifters consume significant amount of DC electrical power making the phased array technology very expensive. On the other hand, the mechanically scanning reflector antennas are widely used for their simplicity, however, these antennas use mechanical rotating device to move the reflector toward the intended signal direction and the spinning speed of the rotary joint used in the reflector antenna constraints the antenna’s agile beamscanning capability. Additionally, the presence of the antenna feed at the front of the reflector creates signal blockage and requires a subreflector to avoid the feed blockage which makes the overall system cost-inefficient and bulky. An alternative to both the mechanically scanning reflector antennas and electronically scanning phased array antenna system is the use of the gradient-index (GRIN) lens-based antenna such as the Luneburg lens antenna. The Luneburg lens is a spherical-shaped graded dielectric structure in which electromagnetic energy from a feed source placed on one surface of the lens radiates as plane wave from the opposite surface of the sphere. This focusing nature of the Luneburg lens allows it to be used as wide-angle beam scanning antenna element for multifunctional wideband antenna applications and beam scanning is simply achieved by switching between an array of antenna feeds placed along the lens’s surface. The benefits include high antenna gain, broad bandwidth, multiple simultaneous beams and reduced electrical power consumption. In particular, the ability to form multiple simultaneous beams without any mechanical moving parts and the presence of the feed source at the back of the antenna make the Luneburg lens an attractive choice to use as wide-angle, multi-beam, multiband antenna applications such as wireless communications and direction finding.

Although the Luneburg lens offers significant potential benefits over phased array systems and reflector antennas to use as a high gain, multibeam multiband antenna element, the lens in its spherical shape is incompatible for practical antenna applications. The spherical geometry of the Luneburg lens structure poses a significant implementation challenge as it complicates the integration of the standard planar feed sources (e.g. waveguides, antenna arrays, detectors) to the lens’s spherical surface. To eliminate this feed mismatch problem between the spherical Luneburg lens and planar feed elements, a more convenient approach is to use a modified Luneburg lens antenna in which the lens’s spherical feed surface is transformed into a planar surface to successfully integrate the lens with planar feed sources and other external back-end electronics^1,3,4^. To design such a modified Luneburg lens antenna while maintaining the spherical lens’s original electromagnetic performances, the recently developed transformation optics method has drawn great interests among the antenna and electromagnetic community^5,6^. Transformation Optics (TO) is an emerging technology which enables to control the propagation of electromagnetic waves inside an electromagnetic structure and has been adopted in many electromagnetic designs^1–59^ including graded-index (GRIN) lenses^1–4,6–29^. TO method employs the form-invariance principle of Maxwell’s equations under coordinate transformation and spatially transforms the constitutive parameters (i.e. permittivities and permeabilities) of an electromagnetic structure which goes through some level of geometric modifications^5^.

However, the material parameters required to implement the transformation optics device are anisotropic with both the electric and magnetic responses (i.e. inhomogeneous permittivity and inhomogeneous permeability materials) and requires the use of resonant metamaterials^5^. To practically realize such a complex anisotropic material, especially the inhomogeneous permeability magnetic material, is very difficult and an implementation challenge. Besides, the requirement of using metamaterials restricts the device functionality to a narrow frequency band. To alleviate the implementation problem associated with the anisotropic TO-based materials, an approximated concept of transformation optics, called quasi-conformal transformation optics (QCTO), was introduced^6^. In the QCTO approximation, the anisotropic nature of the TO-based medium is eliminated by approximating the material’s magnetic responses (i.e. inhomogeneous permeability) to that of free space (i.e. µ_r_ = 1) while retaining the material’s electric responses (i.e. positive inhomogeneous permittivity material) to achieve the same functionality. The advantages include the reduction of the anisotropic medium into an isotropic medium which makes the device implementation much easier and the absence of resonant metamaterials which allows the practically realized device to operate over broad frequency band. It is worth mentioning that the quasi-conformal mapping is limited in two-dimensional configurations and have been widely adopted in antenna applications^1–4,6–59^. For example, a two-dimensional modified Luneburg lens using QCTO method was demonstrated in the X-band^3^. A method to extend the two-dimensional QCTO method to design three-dimensional electromagnetic structure was proposed^6^ and a three-dimensional realization of QCTO modified Luneburg lens antennas were demonstrated in the Ku-band (12.4–18 GHz) and Ka-band frequency (26–40 GHz)^1,4^.

While the QCTO method offers significant benefits to implement a modified Luneburg lens antenna with a planar feed surface using all-dielectric materials, this technique is offset by the presence of reflections^6^ and these reflections arise from the permittivity mismatches between the lens’s planar feed surface and air. In the original transformation optics method, the transformation medium requires both electric and magnetic responses, and the magnetic responses ensure uniform impedance matching and polarization independence. As the magnetic responses are absent in the QCTO method to make the device realization easier, the reflections are simply generated by the electric responses, i.e. inhomogeneous permittivity, achieved in quasi-conformal mapping. To minimize the reflections of the QCTO modified Luneburg lens and improve the antenna gain over a broad frequency band, the feed sources of the modified lens antenna need to be designed very carefully, e.g. implementing dielectrically loaded antenna feed array, to ensure uniform impedance matching and high gain. However, implementing an array of dielectrically loaded antenna feeds will be very difficult due to the inhomogeneous nature of the antenna feed surface’s permittivity profile.

In this work, we show that the reflections present in QCTO method can be mitigated by incorporating a broadband anti-reflective (AR) layer along with the QCTO modified Luneburg lens’s planar excitation surface. Here, we designed a broadband anti-reflective layer and implemented with the modified lens antenna. Like the Luneburg lens structure, the AR layer has a graded-index (GRIN) permittivity distribution which minimizes the impedance mismatches between the QCTO lens antenna and feed sources. The AR layer enabled modified Luneburg lens antenna shows a high aperture efficiency while maintain a relatively high degree of beam scanning over a broad frequency band. To demonstrate the broadband anti-reflective layer enabled new QCTO Luneburg lens design methodology, an example modified lens antenna was designed using quasi-conformal mapping and implemented with the broadband anti-reflective layer to operate in the Ka-band (26–40 GHz). The modified lens antenna was realized using the fused deposition modeling (FDM) based additive manufacturing technique^4^ to operate in the Ka-band frequency range (26–40 GHz). The electromagnetic performance of the fabricated lens antenna was experimentally measured and compared with the simulated predictions. Through the design of the broadband anti-reflective layer, we were able to mitigate the impedance mismatches present in QCTO modified Luneburg lens antenna and improve the antenna gain values significantly at most of the feed locations along the QCTO modified Luneburg lens’s planar excitation surface.

## Results

### QCTO modified Luneburg lens antenna design

**Figure 1:**
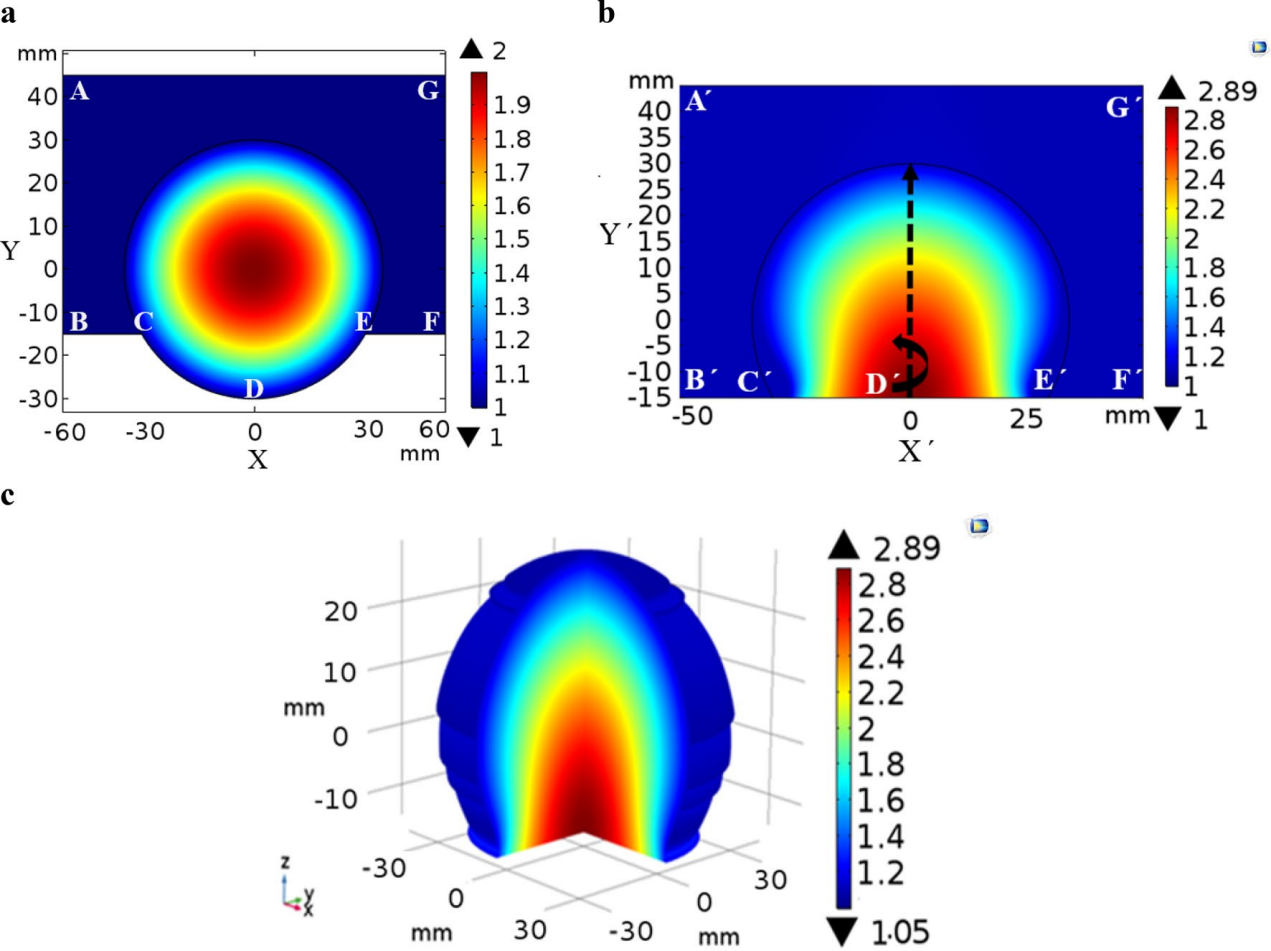
2D QCTO Luneburg lens and 3D-approximate QCTO Luneburg lens design. (**a**) Permittivity profile of the two-dimensional original Luneburg lens. Lens has a radius of 30 mm. (**b**) Permittivity distribution of the two-dimensional QCTO modified Luneburg lens. (**c**) Axisymetrically rotated three-dimensional QCTO modified Luneburg lens’s permittivity distribution. The 3D Lens has an overall dimension of 60 mm × 60 mm × 45 mm whereas the flat-bottom surface has a dimension of 50 mm × 50 mm.

The Luneburg lens is a spherical shaped graded-index (GRIN) dielectric structure in which the dielectric permittivity values changes radially, expressed as ${\varepsilon }_{r}=2-{\left(r/a\right)}^{2}$; where r is the radial distance from the center of the lens, and a is the radius of the sphere^60^. To design a modified Luneburg lens antenna, where portion of the lens’s spherical feed surface is transformed into a flat surface, the quasi-conformal transformation optics (QCTO) method was utilized. As QCTO method is restricted in two-dimensional geometry, here, we conducted a quasi-conformal mapping of the two-dimensional Luneburg lens to modify it into a two-dimensional flat-bottom lens. Figure 1a represents the virtual space, a two-dimensional spherical Luneburg lens structure surrounded by the air and Fig. 1b is the physical space representing the two-dimensional modified Luneburg lens. The two lenses have the same boundaries except the region CDE and C′D′E′. The boundary CDE in virtual space is quasi-conformally mapped into the region C′D′E′ in physical space and the mapping was performed in the physical space by solving the inverse Laplace’s equations subject to Dirichlet–Neumann boundary conditions:

$$\left. {A^{\prime}B^{\prime}} \right|_{{x^{\prime}}} = \left. {F^{\prime}G^{\prime}} \right|_{{x^{\prime}}} = x;\,\, \left. {C^{\prime}D^{\prime}E^{\prime}} \right|_{{x^{\prime}}} = 1.85\pi \cdot \frac{x}{2};\quad \hat{n} \cdot \left. {\nabla x} \right|_{{A^{\prime}G^{\prime},\,\, B^{\prime}C^{\prime},E^{\prime}F^{\prime}}} = 0$$

$$\left. {A^{\prime}G^{\prime}} \right|_{{y^{\prime}}} = \left. {B^{\prime}C^{\prime}} \right|_{{y^{\prime}}} = \left. {E^{\prime}F^{\prime}} \right|_{{y^{\prime}}} = y;\,\, \left. {C^{\prime}D^{\prime}E^{\prime}} \right|_{{x^{\prime}}} = - \sqrt {R^{2} - x^{2} } ;\quad \hat{n} \cdot \left. {\nabla y} \right|_{{A^{\prime}B^{\prime}, \,\, F^{\prime}G^{\prime}}} = 0$$

The QCTO mapping was carried out numerically using the COMSOL Multiphysics solver and the new material parameters of the modified two-dimensional Luneburg lens were calculated as:

$${\varepsilon }^{^{\prime}}=\frac{{\varepsilon }_{r} }{\left|{\boldsymbol{\Lambda }}^{-1}\right| };\quad {\mu }^{{\prime}}=1$$

Here, ***Λ*** is the Jacobian transformation matrix which relates the coordinate transformation between the physical space and the virtual space. Figure 1a shows the dielectric permittivity distribution of the two-dimensional original Luneburg lens and Fig. 1b presents the calculated permittivity profile of the modified Luneburg lens antenna. The three-dimensional realization of the permittivity profile (Fig. 1c) was achieved by axisymetrically revolving the two-dimensional profile (Fig. 1b) along its center axis.

**Figure 2:**
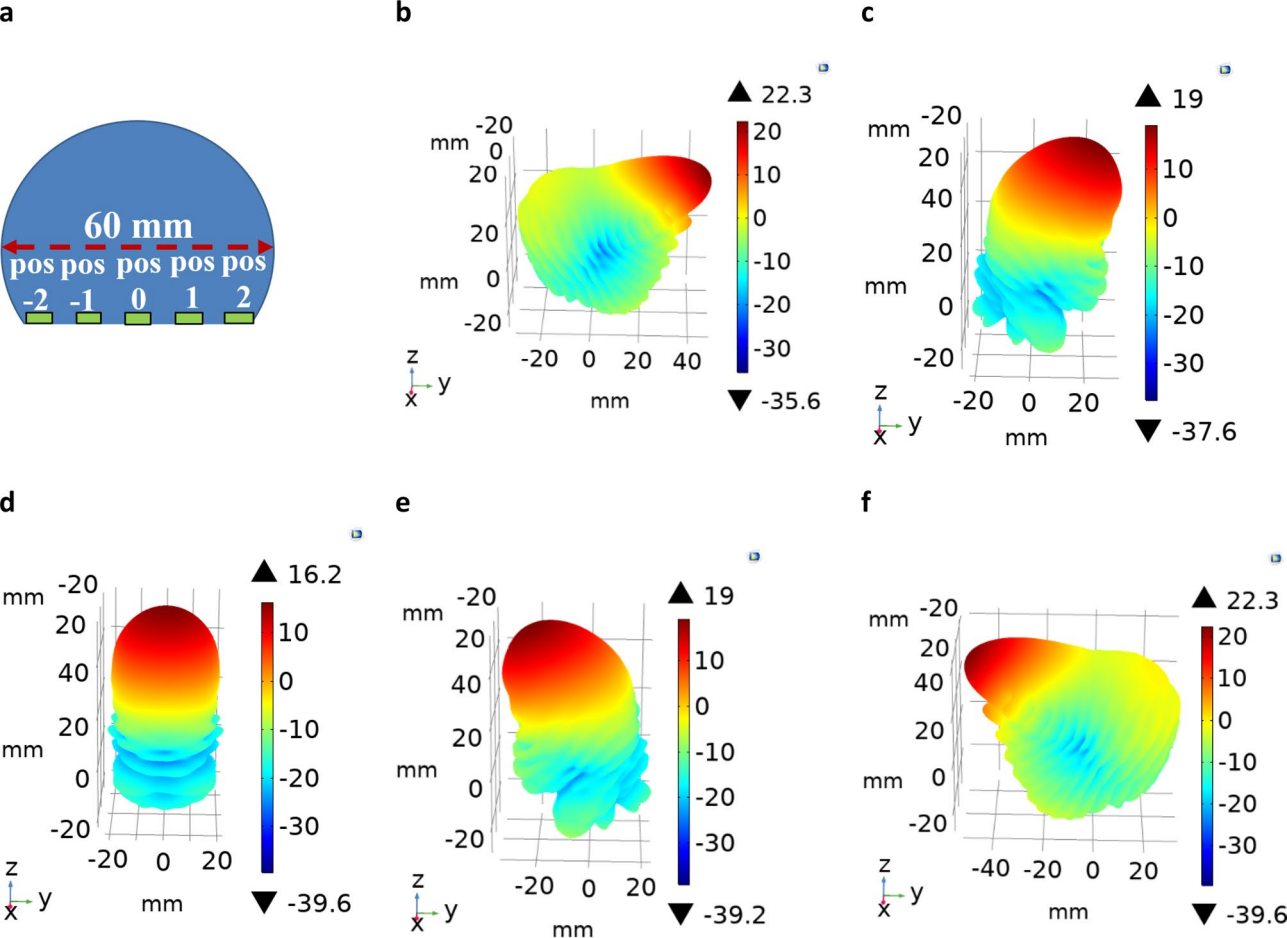
Far-field radiation patterns of the QCTO modified Luneburg lens antenna at 30 GHz frequency. (**a**) Lens’s geometry indicating the five feed locations to which a single open-ended- waveguide port was moved along a center line of the planar feed surface. Gain patterns at (**b**) pos − 2 (+ 55°); (**c**) pos − 1 (+ 22°); (**d**) pos 0 (0°); **e),** pos 1 (− 22°); f) pos 2 (− 55°).

To demonstrate the electromagnetic performance of the modified Luneburg lens antenna (Fig. 1c), full-wave electromagnetic simulations were conducted, and the lens’s gain patterns and beam-steering angle were calculated at Ka-band (26–40 GHz). To excite the lens, an open-ended waveguide feed was placed at the lens’s flat-bottom surface and the waveguide feed was mechanically moved along the planar surface. To show the beam-steering angle and gain patterns, the lens antenna was excited at five feed locations as shown in Fig. 2a and at each location, the lens’s radiation patterns were calculated using COMSOL™ numerical solver. Figure 2b–f represents the calculated 3D radiation patterns at 30 GHz for the five waveguide feed sources. From the simulated results it is observed that, the lens showed a relatively wide beamscanning angle (from − 55° to + 55°) with a continuously reduced gain value as the feed moves from the outward edges to the center.

**Figure 3:**
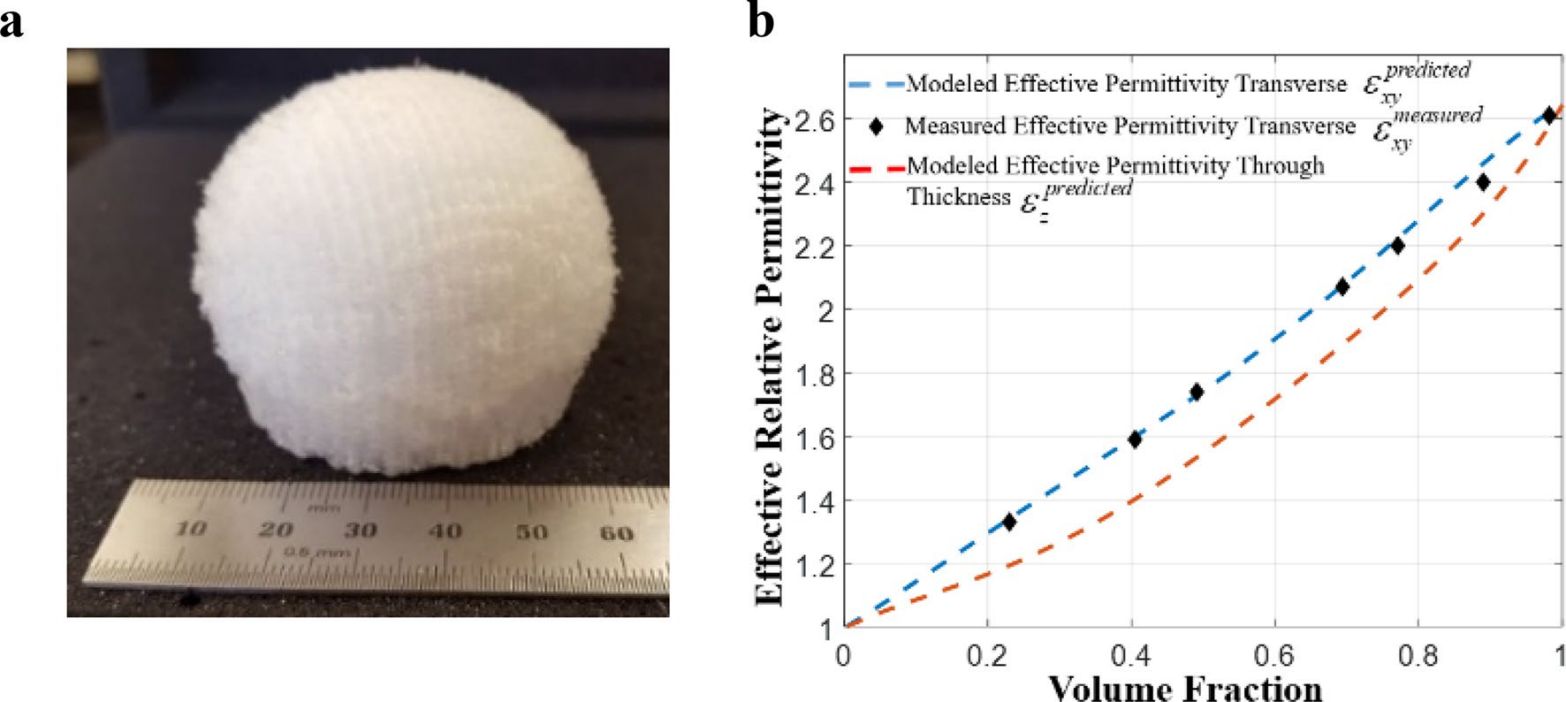
Additively manufactured graded-index (GRIN) QCTO Luneburg lens structure. (**a**) Fabricated QCTO-enabled modified Luneburg lens antenna; (**b**) Permittivity variation of printed polymer with volume fraction of the material to air.

The modified Luneburg antenna designed with quasi-conformal mapping was practically realized using fused deposition modeling (FDM) based additive manufacturing (AM) technique^4,61^. Here, AM is used to generate small scale changes (i.e. less than wavelength) in polymer which results in an effective local permittivity as a function of the local volume fraction of printed material to air. By changing the material volume fraction spatially, the graded permittivity distribution of the QCTO modified Luneburg lens was realized. Figure 3a presents the fabricated QCTO-enabled flat-bottom Luneburg lens antenna and Fig. 3b shows the permittivity variation as a function of volume fraction of the dielectric material. The details description of the fabrication method is described in Ref.^4^.

**Figure 4:**
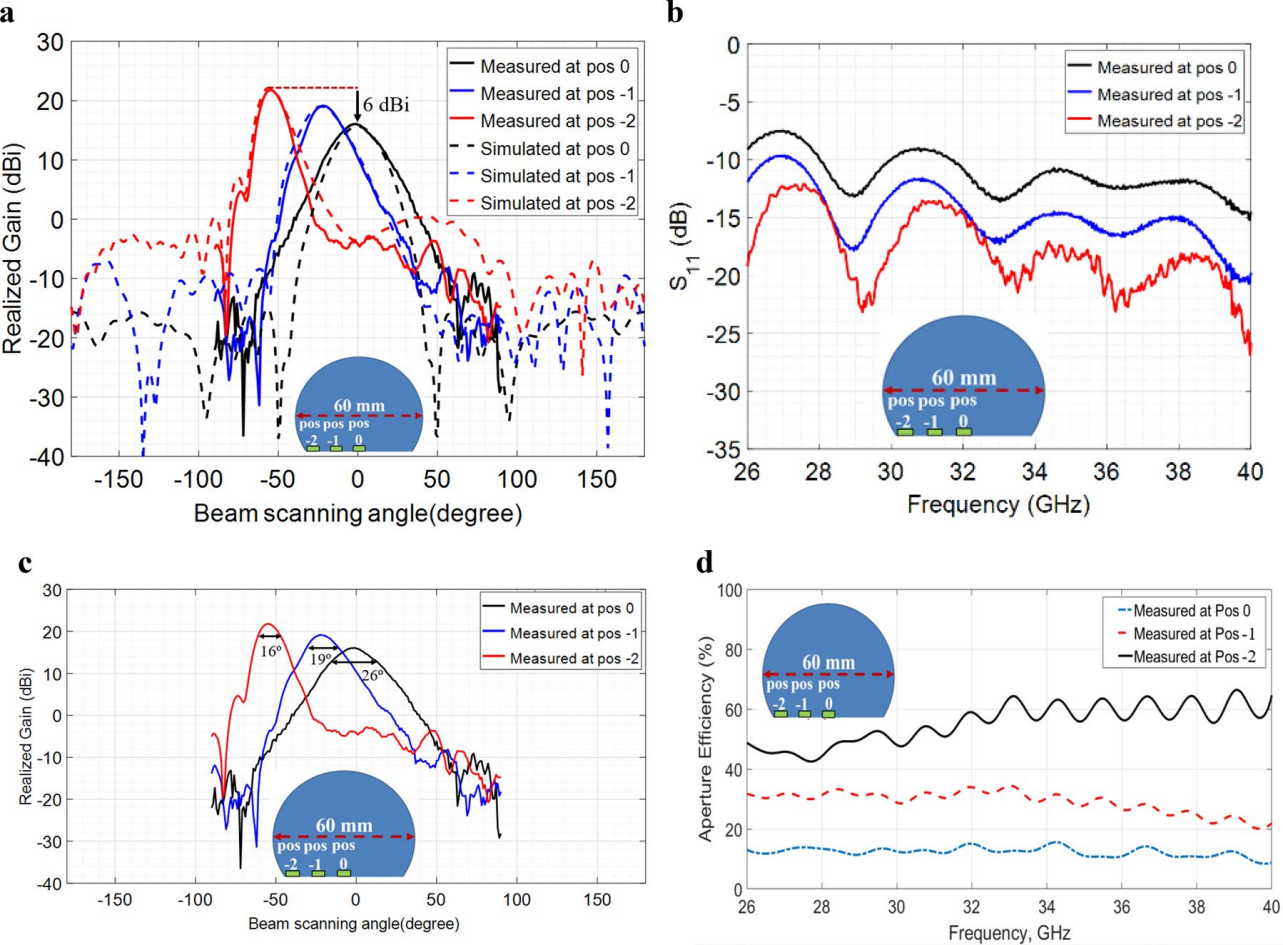
Measured results of the QCTO modified Luneburg lens antenna. (**a**) Measured and simulated gain patterns at 30 GHz as a function of beam steering angle; (**b**) Measured return loss (S_11_); (**c**) Measured Half-power (3 dB) beamwidth at 30 GHz for three feed locations (pos − 2, pos − 1, pos 0); (**d**) Measured aperture efficiencies of the lens at Ka-band for three feed locations (pos − 2, pos − 1, pos 0).

To compare the electromagnetic performance of the fabricated modified lens antenna with the simulated predictions, the lens antenna was experimentally characterized by measuring the mismatch losses and the gain patterns as a function of frequency, beam scanning angle and location of the feed source. An open-ended waveguide (WR28) was used as a feed source in the measurement and the waveguide feed was mechanically moved along the planar surface from the outward edge toward the center of the lens. Due to the rotational symmetry of the lens antenna, we are presenting the results at only three feed locations here. Figure 4a compares the simulated and measured gain patterns as a function of beam scanning angle at 30 GHz for the three feed locations (pos − 2, pos − 1, pos 0). It is obvious that the lens showed similar beam-steering performances (0° to − 55°) as the simulated predictions. The rotational symmetry of the Luneburg lens allows similar performance from 0° to + 55°. However, from the measurement and simulation, it is also clear that the quasi-conformally mapped modified Luneburg lens antenna showed a continuous reduction in the maximum gain value as the waveguide feed was moved from the outward edge (pos − 2) toward the center (pos 0) of the lens. This is because of the mismatch losses present in quasi-conformal mapping and the losses are generated by the permittivity mismatches between the modified lens’s planar feed surface and free space. As the permittivity mismatch was highest at the center of the lens (ε_lens_ = 2.89 and ε_air_ = 1), the reflections were also greater at that location resulting in the lowest gain value. As the permittivity values gradually become lower from the center of the lens toward the outward edges, the mismatch losses start minimizing which increases the gain value. The lens antenna showed a highest gain value at the outward edge (pos − 2) where the permittivity value of the lens’s planar surface is closer to the air. Figure 4b presents the measured reflection coefficients (S_11_) of the QCTO modified Luneburg lens antenna for the three open-ended waveguide feeds and the free-space waveguide losses without the inclusion of the lens.

In addition to the mismatch losses present in QCTO mapping, it was also observed that the interaction between the higher dielectric constant and the waveguide feed signal results in wider half-power (3 dB) beamwidth which further reduces the lens’s overall gain value significantly. Since, the modified lens antenna had a highest permittivity value at the center (pos 0), the half-power beamwidth became wider at that location (26°) and the beamwidth was lowest at the edge (16°) where the dielectric constant was nearer to the air. Figure 4c demonstrates the measured half-power (3 dB) beamwidth of the QCTO modified Luneburg lens’s radiation patterns at three feed locations (pos − 2, pos − 1, pos 0). Because of the mismatch losses and beam widening, the modified lens antenna showed a lower aperture efficiency (less than 20%) at the center feed location (pos 0) and the aperture efficiency gradually became higher as the feed moved toward the edge (pos − 2) from the center (pos 0). The lens antenna had a highest aperture efficiency of more than 50% at the edge. Figure 4d demonstrates the measured aperture efficiencies of the modified Luneburg lens antenna at three feed locations over the entire Ka-band (26–40 GHz).

### Broadband anti-reflective (AR) layer with QCTO modified Luneburg lens

**Figure 5:**
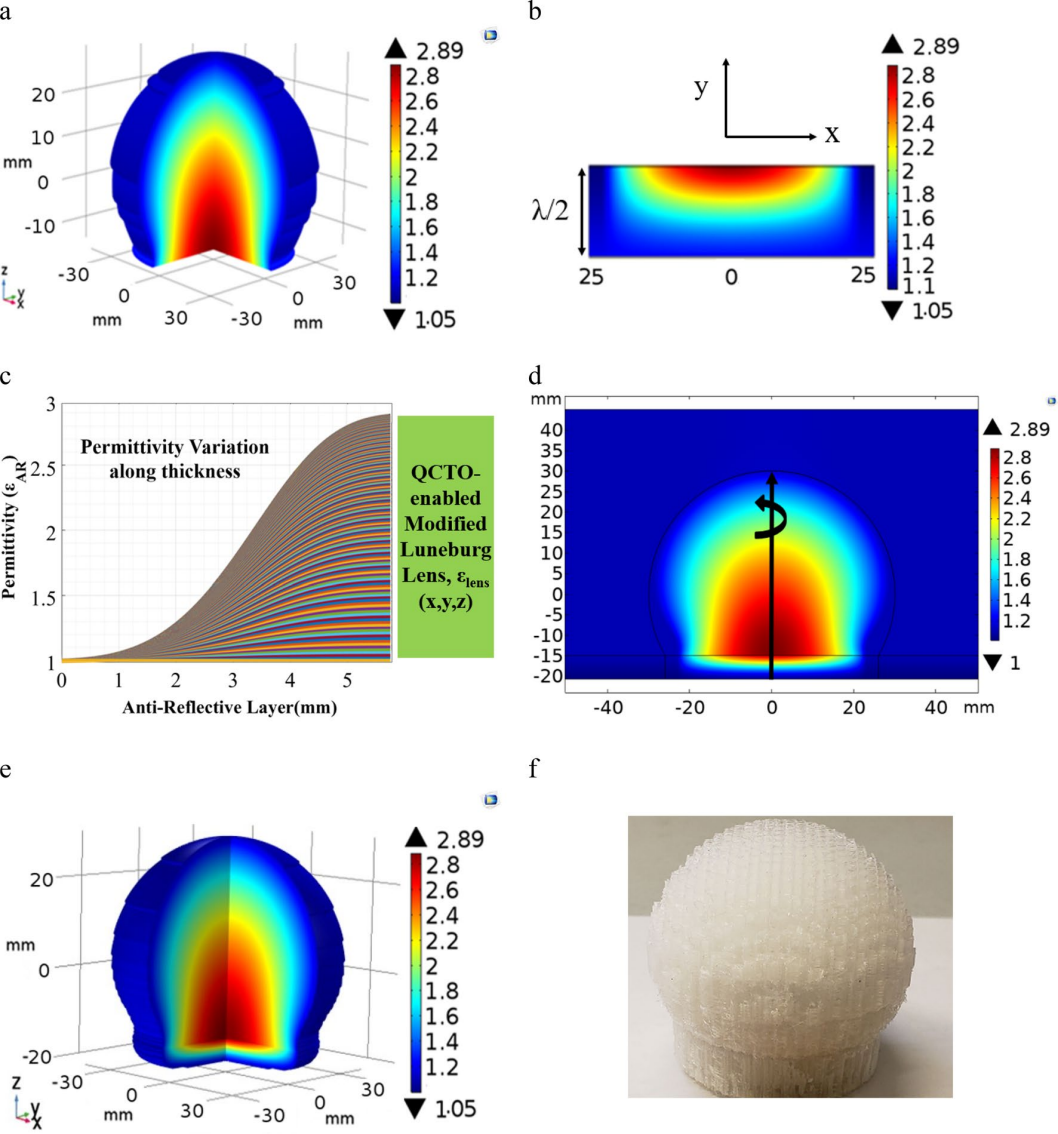
Broadband anti-reflective (AR) layer along with QCTO modified Luneburg lens. (**a**) QCTO-modified flat-bottom Luneburg lens antenna; (**b**) two-dimensional inhomogeneous permittivity profile of the λ_26GHz_/2 long anti-reflective layer; (**c**) Graphical representation of the AR layer’s permittivity vairation along its thickness; (**d**) two-dimensional permittivity profile of the AR layer incorporated QCTO modified Luneburg lens antenna; (**e**) axisymmetrically rotated three-dimensional permittivity distribution of the flat-bottom Luneburg lens with broadband anti-reflective layer. The lens has a physical dimension of 60 mm × 60 mm × 50.76 mm. whereas the bottom planar surface has a dimension of 50 mm x 50 mm; (**e**) additively fabricated QCTO modified Luneburg lens antenna with the broadband anti-reflective layer.

The mismatch losses and half-power beamwidth broadening problem present in QCTO modified Luneburg lens antenna can be mitigated by incorporating an anti-reflective (AR) layer between the modified lens’s planar surface (Fig. 5a) and free space. A quarter-wave long anti-reflective layer is the simplest way to counter the mismatch losses; however, this technique will limit the lens’s functionality to only narrow frequency bands. To minimize the mismatches over broad frequency bands and increase the lens’s gain value at all feed locations, we designed and implemented a broadband anti-reflective layer in conjunction with the QCTO modified Luneburg lens antenna. Like the Luneburg lens, the designed anti-reflective layer has an inhomogeneous permittivity profile which tapers the permittivity values of the modified lens’s flat-bottom surface to that of free space and mitigates the impedance mismatches over a broad frequency band at all feed locations across the planar surface. Besides, the AR layer narrows the half power (3 dB) beamwidth and further increase the lens’s gain value. To design such a broadband anti-reflective layer with a continuously tapered permittivity distribution, we explored the Klopfenstein impedance mitigation method described for microwave transmission lines and implemented the Klopfenstein tapered impedance profile in context of the QCTO modified Luneburg lens’s permittivity distribution. A Klopfenstein profile describes the changes in the characteristic impedances along a microwave transmission line with length L, expressed as^62^

$$\mathrm{ln}\,\, Z\,\,\left(z\right)=\frac{1}{2}{\mathrm{ln\, Z}}_{0}\,\,{\mathrm{Z}}_{\mathrm{L}}+\frac{{\Gamma }_{0}}{\mathrm{cos h}\,A} {\mathrm{A}}^{2}\Phi \left(2\frac{\mathrm{z}}{\mathrm{L}}-1,\mathrm{ A}\right);\quad \mathrm{for}\,\, 0\le \mathrm{z}\le \mathrm{L}$$

And the permittivity values required to mimic the Klopfenstein characteristic impedance along the AR layer’s thickness L can be expressed as^58,62,63^:

$${\upvarepsilon }_{\mathrm{AR}}(z)={\upvarepsilon }_{\mathrm{i}}{\upvarepsilon }_{\mathrm{s}}\,\,\mathrm{exp}\left[{2\Gamma }_{\mathrm{m}} {\mathrm{A}}^{2}\Phi \left(2\frac{\mathrm{z}}{\mathrm{L}}-1,\mathrm{ A}\right) \right];\quad {\mathrm{ for }}\,\,0\le \mathrm{z}\le \mathrm{L}$$

Here, ε_s_ represents the inhomogeneous permittivity profile of the modified Luneburg lens’s flat-bottom surface (Fig. 5a) and ε_i_ is the permittivity value of air (ε_r_ = 1). L is the optimum thickness of the anti-reflective layer required to minimize the mismatch losses. The function Φ (x, A) is defined as

$$\Phi \left(\mathrm{x},\mathrm{ A}\right)=-\Phi \left(-\mathrm{x},\mathrm{ A}\right)={\int }_{0}^{\mathrm{x}}\frac{{\mathrm{I}}_{1}\left(\mathrm{A}\sqrt{1-{\mathrm{y}}^{2}}\right)}{\mathrm{A}\sqrt{1-{\mathrm{y}}^{2}}}\mathrm{dy};\quad \mathrm{ for }\,\,\left|\mathrm{x}\right|\le 1$$

where *I*_1_ is the first kind modified Bessel function of order one. The maximum reflection (Г_m_) and initial reflection coefficient (Г_0_) are defined as^7,62^:

$${\Gamma }_{\mathrm{m}}=\frac{{\Gamma }_{0}}{\mathrm{cosh\, A}} ;\quad {\Gamma }_{0}=\frac{1}{2}\mathrm{ln}\left(\frac{\sqrt{{\upvarepsilon }_{\mathrm{s}}}}{\sqrt{{\upvarepsilon }_{\mathrm{i}}}}\right)$$

To implement the anti-reflective layer, the two-dimensional Klopfenstein permittivity profile of the AR layer was calculated using the COMSOL-MATLAB interface and incorporated with the two-dimensional QCTO modified Luneburg lens antenna (Fig. 1b). To ensure a uniform impedance matching at all feed locations and maintain a wide beam scanning angle with high gain value, the optimum thickness of AR layer is very crucial. After conducting several parametric studies, it was found that a λ/2 (at lowest frequency) long anti-reflective layer provides the optimum performance of the modified Luneburg lens antenna in terms of high gain and wide beam scanning angle. Figure 5b presents the calculated two-dimensional permittivity profile of the λ/2 long broadband Klopfenstein anti-reflective layer and Fig. 5c shows the changes in all the permittivity values of the QCTO lens’s bottom surface along the thickness of the AR layer. The two-dimensional AR layer was incorporated with the modified two-dimensional Luneburg lens antenna and Fig. 5d demonstrates the two-dimensional permittivity profile of the AR layer incorporated QCTO modified Luneburg lens antenna. The three-dimensional realization of the permittivity distribution (Fig. 5e) was generated by axisymetrically rotating the two-dimensional profile (Fig. 5d) along its center axis. The broadband anti-reflective layer enabled modified Luneburg lens antenna was fabricated using fused deposition modeling (FDM) based additive manufacturing technique as described in the previous section and Fig. 5f represents the fabricated lens antenna with the broadband anti-reflective layer.

**Figure 6:**
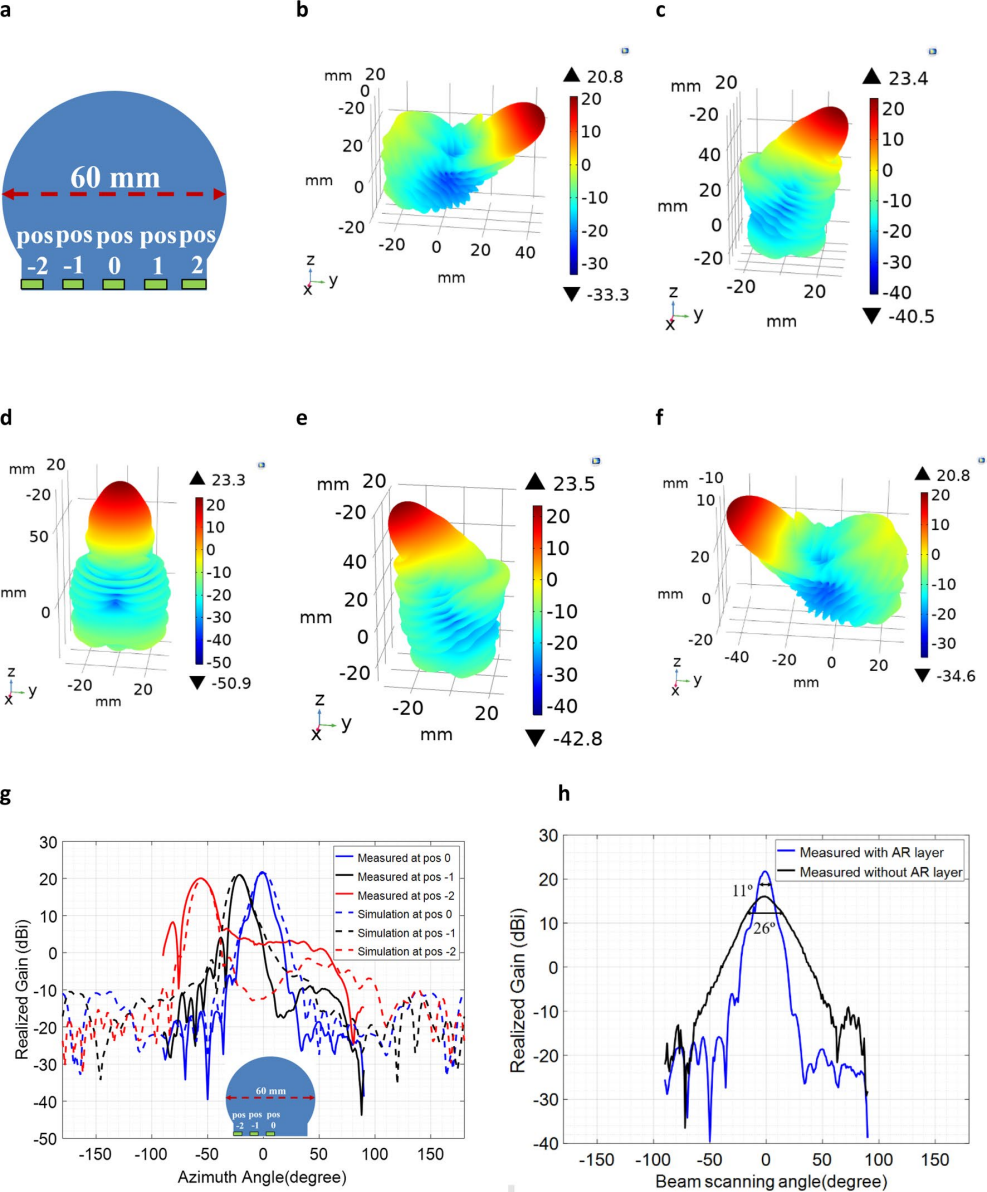
Anti-Reflective layer mitigates the reflection problems present in QCTO method and improves the gain values. Simulated 3D far field radiation patterns of modified Luneburg lens antenna at 30 GHz with the presence of λ/2 (at 26 GHz) thickness anti-reflective layer. lens’s beam-steering angle at (**a**) − 55° (pos − 2); (**b**) − 22° (pos − 1); (**c**) 0° (pos 0); (**d**) 55° (pos 2); **e**) 22˚ (pos 1); (**f**) feed source locations at the planar excitation surface. (**g**) measured and simulated gain patterns comparison at 30 GHz with and without the presence of AR layer at 3 excitation locations (pos − 2, pos − 1, pos 0).

To show the broadband impedance mismatch mitigation and compare the electromagnetic performance, full-wave electromagnetic simulations were conducted at Ka-band using the COMSOL numerical solver. To excite the lens antenna, a waveguide port was used as a feed source at the same five feed locations as used in the QCTO Luneburg lens without the AR layer, along the AR layer’s flat-bottom surface as shown in Fig. 6a. At each location, the far-field radiation pattern was calculated using the COMSOL solver. Figure 6b–f demonstrates the calculated three-dimensional radiation patterns at 30 GHz frequency corresponding to each feed location. From the simulated results, it is evident that the presence of the half-wave long (at 26 GHz) anti-reflective layer mitigates the impedance mismatches and improves the lens’s maximum gain value significantly at most of the feed locations while maintaining the lens’s wide beam scanning angle (± 55°).

The fabricated lens antenna (Fig. 5f) was measured experimentally to compare with the simulated predictions. Figure 6g compares the measured and simulated gain patterns of the AR layer enabled QCTO modified Luneburg lens antenna at 30 GHz frequency at three feed locations (pos -2, pos -1, pos 0). We are presenting the results for three feed locations as the rotational symmetry of the lens antenna allows similar performance at all other feed locations along the entire planar feed surface. From the results, it is obvious that the anti-reflective layer mitigates the mismatch losses present in quasi-conformal mapping and increases the gain value significantly. Additionally, the AR layer’s permittivity profile reduces the half-power (3 dB) beamwidth and increases the lens’ overall gain value. Figure 6h shows the measured half-power (3 dB) beamwidth of the modified lens antenna with and without the presence of AR layer at the center feed location. We are presenting the result for the waveguide feed located at the center as the beamwidth was widest at that location compared to outward edges (Fig. 4c).

Even though the anti-reflective layer mitigates the reflections present in quasi-conformal mapping and increases the lens’ gain value at most of the feed positions, the incorporation of the anti-reflective layer with the QCTO modified Luneburg lens antenna reduces the lens’s maximum gain value at the outward edge feed location (pos − 2). This is because the modified lens antenna without the presence of the AR layer (Fig. 1c) had a minimum permittivity mismatch at the outward edges which resulted in maximum gain value at the edges (pos − 2). As the anti-reflective layer is included with the lens antenna to mitigate the mismatch losses at most of the feed locations (pos − 1, pos 0), the focal point of the lens located at the outward edge (pos − 2) moves away by the AR layer’s thickness and the shift in the focal point at the edge results in scattering and side lobes with a lower gain value.

**Figure 7:**
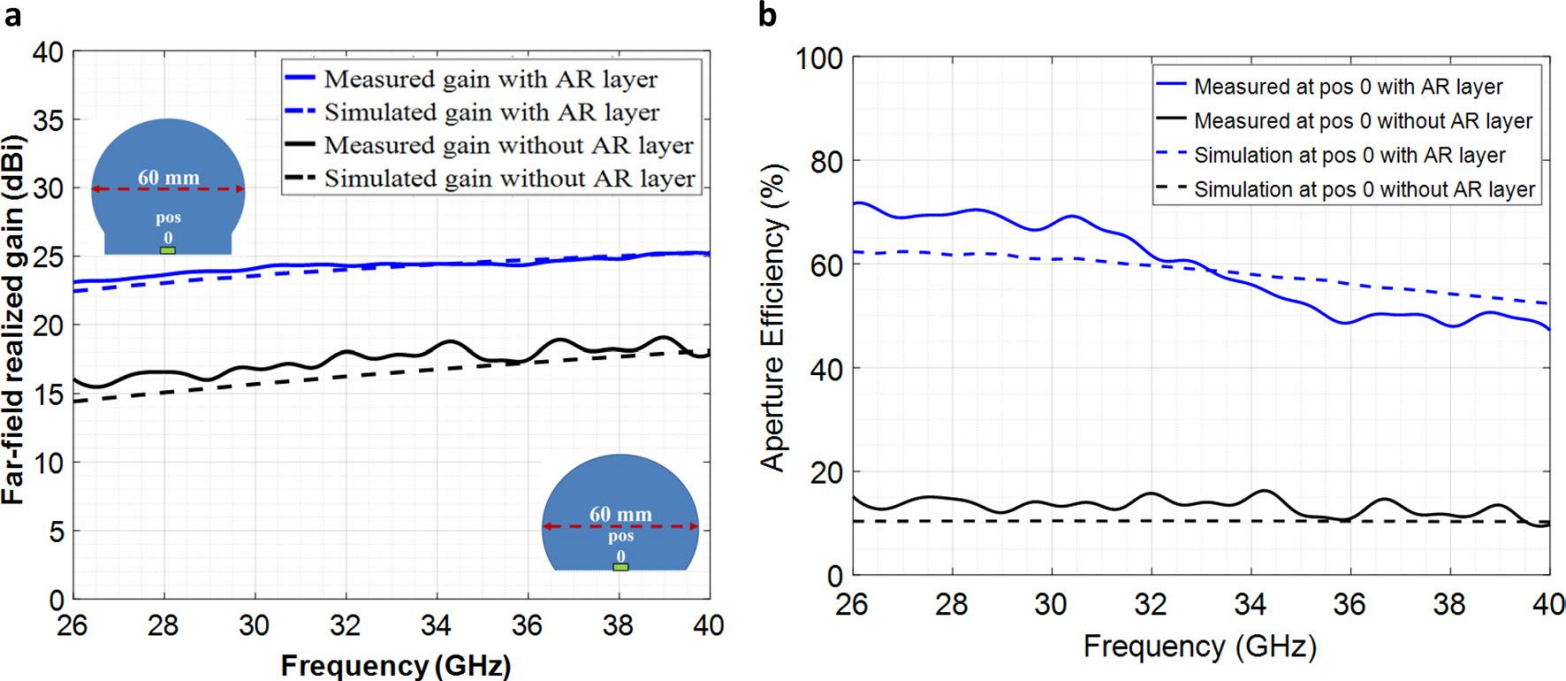
Broadband performance of the anti-reflective layer. (**a**) Measured and simulated far-field gain patterns of the QCTO Luneburg lens with and without the presence of AR layer at Ka-band. (**b**) Measured and simulated aperture efficiencies as a function of frequency for the waveguide feed located at the center of the lens (pos 0) with and without the presence of the anti-reflective layer.

To show the broadband nature of the designed anti-reflective layer, we measured the gain patterns over the entire Ka-band (26–40 GHz). Figure 7a compares the measured and simulated gain patterns of the lens antennas (i.e. with and without the presence of AR layer) as a function of frequency. For brevity, the results for the center feed location are presented here. From Fig. 7a it is obvious that the designed anti-reflective layer has a broadband impedance mitigation and increases the lens’s gain value over the entire frequency band. These results are consistent at most of the feed locations across the lens’s planar excitation surface except for the outward edges where the lens showed a reduced gain value. Figure 7b compares the measured aperture efficiencies of the modified lens antennas for the feed source located at the center of the lenses over the entire Ka-band. It is evident that the incorporation of the broadband anti-reflective layer with the QCTO modified Luneburg lens antenna enhances the lens’s aperture efficiency significantly from less than 20% to about 60% at the center feed location.

### Anti-Reflective layer’s thickness effect on lens’s beam-steering performance

**Figure 8:**
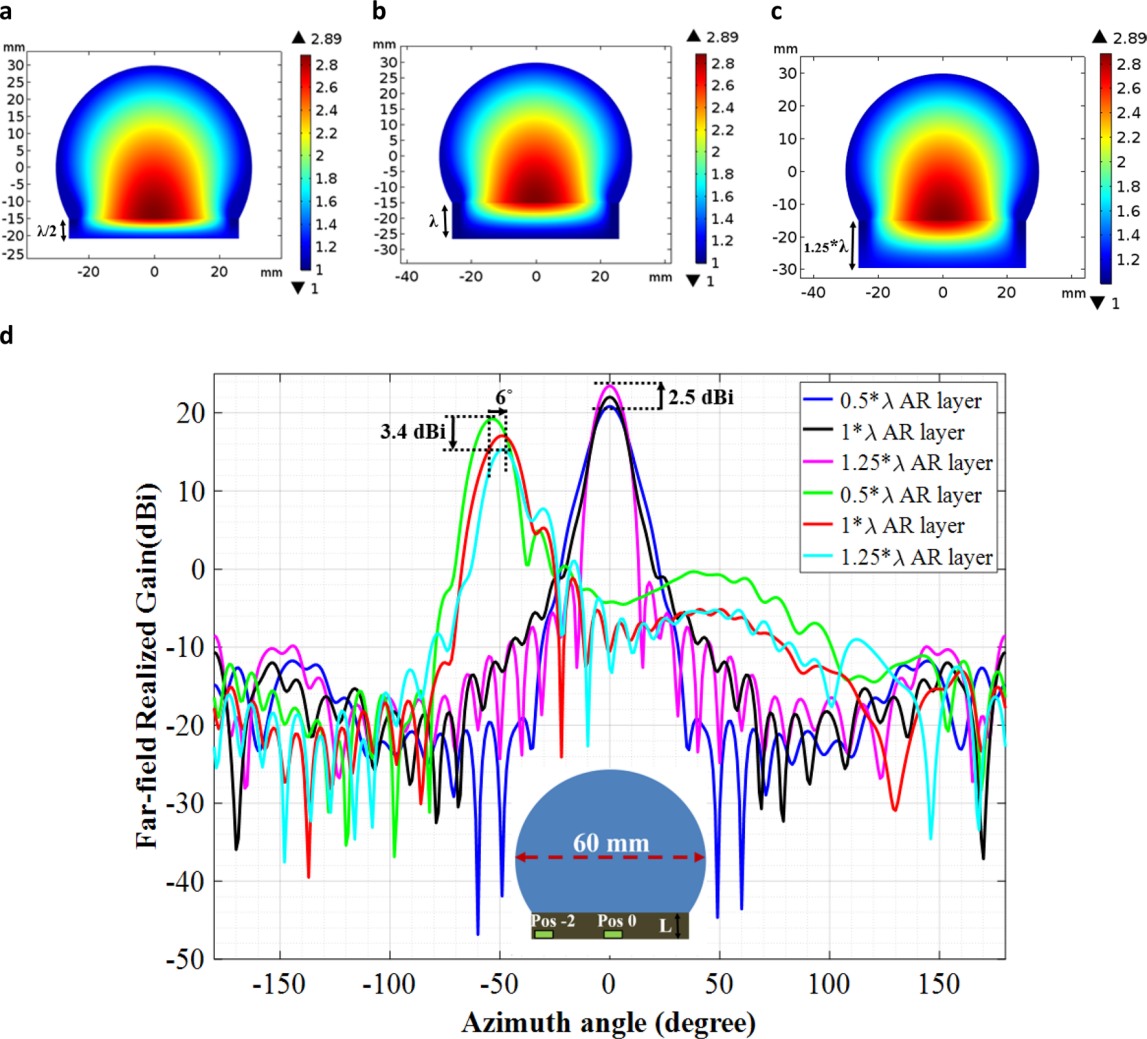
Anti-reflective layer’s thickness effect on lens’s beam-scanning performances and gain value. Cross section view of the permittivity profile of modified Luneburg lens with: (**a**) λ/2 thick AR layer; (**b**) λ thick AR layer; **(c**) 1.25*λ thick AR layer. (**d**) Len’s beam scanning performance and gain patterns for waveguide feed located at the outward edges (pos − 2) and center of the lens (pos 0).

The incorporation of the broadband anti-reflective (AR) layer does mitigate the impedance mismatches and increases the gain value at most of the excitation locations; however, a higher thickness (greater than λ/2) AR layer reduces the lens’s wide beam-scanning angle. For example, in Fig. 8, we designed the QCTO modified Luneburg lens antenna with three different thickness AR layer (i.e. L = λ/2, λ, 1.25*λ). Figure 8(a-c) demonstrates the cross-section view of the permittivity distribution of the lens antenna with anti-reflective layer. Each antenna was excited with a waveguide port at two feed locations and at each location, the gain patterns were calculated at 30 GHz. Figure 8d compares the simulated gain patterns of each of the three lens antennas at two feed locations (pos -2 and pos 0) as a function of the beam-scanning angle. From the simulated results, it is observed that with the increase of the AR layer’s thickness (from λ/2 to 1.25*λ), the lens’s maximum gain value increases significantly (2.5 dBi) for the feed location at the center (pos 0). This is because with the increase of the AR layer thickness, the impedance matching becomes more gradual. However, an increase in the AR layer’s thickness does reduce the lens’s maximum beam scanning angle (6° in this design) when the waveguide feed is located at the outward edges (pos − 2). This is due to the fact that like the Luneburg lens antenna, the anti-reflective layer has a graded-index (GRIN) dielectric profile and with an increase in the thickness, the AR layer starts behaving like a GRIN lens resulting in a reduction in wide beamscanning angle. Also, a higher thickness AR layer does reduce the lens’s maximum gain value at the outward edges (3.4 dBi in Fig. 8d). This is because, an increase in the AR layer’s thickness moves the feed source away from the focal point at the outward edge. But the anti-reflective layer improves the lens’s high gain value and wide beam scanning angle at other feed locations.

## Conclusion

In this work, we have showed the design and implementation of a flat-bottom modified Luneburg lens antenna for practical beam scanning antenna applications. The spherical Luneburg lens antenna was modified into a flat-bottom antenna using the quasi-conformal transformation optics (QCTO) method. The modified lens antenna designed with the quasi-conformal mapping suffered from reflection problem due to the permittivity mismatches between the lens’s feed surface and air. Additionally, the interaction of the incident wave on the higher permittivity feed location widens the lens’s half-power (3 dB) beamwidth resulting in further reduction in gain value and aperture efficiency. The broadband anti-reflective layer used in this design mitigates the impedance mismatches significantly over the entire Ka-band and narrows the lens’s half-power (3 dB) beamwidth at the feed location where the permittivity value is higher than the free space.

The only drawback of the design method is that if the anti-reflective layer becomes longer than λ/2 (at lowest frequency), the device’s maximum beam scanning angle starts reducing due to the gradient-index (GRIN) nature of the anti-reflective layer. Also, a higher thickness AR layer reduces the lens’s maximum gain value at the outward edges due to the shift in the lens’s focal point at those locations. However, the anti-reflective layer does improve the lens’s gain value at most of the feed locations except the edges indicating a broadband impedance mitigation at the lens’s planar feed surface. The anti-reflective layer enabled QCTO modified Luneburg lens design method was validated by designing and fabricating a lens antenna designed to operate at Ka frequency band (26–40 GHz). From the measurement and simulated predictions, it was observed that the anti-reflective layer incorporated QCTO modified Luneburg lens antenna showed a wide-angle beam scanning (-55º to + 55º) with high gain value at most of the feed locations. The lens antenna showed a relatively high aperture efficiency at most feed locations compared to the QCTO Luneburg lens antenna without an anti-reflective layer. Implementing the broadband AR layer with the QCTO modified Luneburg lens antenna resulted in an aperture efficiency of more than 60% at most of the feed locations except at the outward edges. The lens antenna showed an aperture efficiency of about 40% at the extreme outward edges due to the shift in lens’s focal point.

In this work, we showed the broadband impedance mitigation method to compensate the reflections present in the quasi-conformally mapped electromagnetic structure and implemented it in the context of the QCTO-enabled gradient-index (GRIN) modified Luneburg lens antenna. We expect that this anti-reflective layer-based new QCTO device design methodology will have practical applicability and can be extended to many other areas of electromagnetic designs^28–53^ which make use of the quasi-conformal transformation optics (QCTO) method. The incorporation of the broadband anti-reflective layer along with the QCTO designs will improve the device electromagnetic performance significantly and become a robust alternative to the conventional QCTO-based designs which suffer from reflections. Here, we demonstrated the calculation of the proposed anti-reflective layer’s permittivity distribution based on the Klopfenstein impedance taper profile. However, we believe, many other continuously tapered permittivity profiles such as Exponential profile or Gaussian profile can also be exploited to calculate the AR layer’s inhomogeneous permittivity distributions.

## Methods

The numerical simulations such as the 2D QCTO mapping was performed by solving the Laplace’s equations using COMSOL Mathematics module. The calculation of the QCTO permittivity distribution and the first order partial derivatives of the Jacobian transformation matrix was achieved via the COMSOL solver. The calculation and implementation of the anti-reflective layer was performed by using the COMSOL-MATLAB Livelink interface. The 3D full-wave electromagnetic performance of the 3D Luneburg lens antenna was evaluated using COMSOL 3D RF module.

The antenna gain patterns were measured by using an Agilent PNA E83684B vector network analyzer. Here, the transmission coefficients of the lens antenna with respect to a fixed standard gain horn antenna was measured from − 90° to + 90° in 1° increment. To minimize the reflections in the measurement, radar absorbing material was used surrounding the measurement system.
