# Author Correction: High gain, wide-angle QCTO-enabled modified Luneburg lens antenna with broadband anti-reflective layer

**DOI:** 10.1038/s41598-020-77618-6

**Published:** 2021-03-02

**Authors:** Soumitra Biswas, Mark Mirotznik

**Affiliations:** 1 Electrical and Computer Engineering Department, University of Delaware, Newark, DE 19716 USA; 2 Advanced Antenna Technology Group, Envistacom, Peachtree Corners, GA 30092 USA

Correction to: *Scientific Reports* 10.1038/s41598-020-69631-6, published online 28 July 2020

This Article contains errors in the Results section, “QCTO modified Luneberg lens antenna design”, there is an error in first line of the given equation.

$${\left.{A}^{{\prime}}{B}^{{\prime}}\right|}_{{x}^{{\prime}}}={\left.{F}^{{\prime}}{G}^{{\prime}}\right|}_{{x}^{{\prime}}}=x ; {\left.{C}^{{\prime}}{D}^{{\prime}}{E}^{{\prime}}\right|}_{{x}^{{\prime}}}=1.85\pi \cdot x/2; \;\widehat{n}\cdot {\left.\nabla x\right|}_{{A}^{{\prime}}{G}^{{\prime}}, {B}^{{\prime}}{C}^{{\prime}},{E}^{{\prime}}{F}^{{\prime}}}=0$$

should read:

$${\left.{A}^{{\prime}}{B}^{{\prime}}\right|}_{{x}^{{\prime}}}={\left.{F}^{{\prime}}{G}^{{\prime}}\right|}_{{x}^{{\prime}}}=x ; {\left.{C}^{{\prime}}{D}^{{\prime}}{E}^{{\prime}}\right|}_{{x}^{{\prime}}}=1.85\cdot \frac{\pi }{3}\cdot x/2;\; \widehat{n}\cdot {\left.\nabla x\right|}_{{A}^{{\prime}}{G}^{{\prime}}, {B}^{{\prime}}{C}^{{\prime}},{E}^{{\prime}}{F}^{{\prime}}}=0$$

